# Supplementary material for: Electrical manipulation of skyrmions in a chiral magnet
Source: Nat Commun. 2022 Mar 24;13:1593. doi: 10.1038/s41467-022-29217-4 (PMC8948302; doi:10.1038/s41467-022-29217-4)
Supplement: Supplementary file 1 — Description of Additional Supplementary Files [file 41467_2022_29217_MOESM1_ESM.pdf]

Title: Supplementary Movie 1:
Description: The procedure of the Co8Zn10Mn2 micro-device fabrication for in-situ Lorentz TEM experiments using the FIB-SEM dual-beam system.

Title: Supplementary Movie 2:
Description: The movie contains the raw images shown in Fig. 1a, in which the skyrmion creation process after applying the current pulses can be found.

Title: Supplementary Movie 3:
Description: The movie contains the raw images shown in Supplementary Fig. S3, in which the skyrmion creation for both negative and positive currents in the thermal-dominated region can be found.

Title: Supplementary Movie 4:
Description: The movie contains the raw images shown in Fig. 2a, in which the skyrmion deletion after applying the current pulses can be found.

Title: Supplementary Movie 5:
Description: The movie contains the raw images shown in Fig. 3a, in which a single skyrmion with Q = -1 is moved using current pulses.

Title: Supplementary Movie 6:
The movie contains the raw images shown in Fig. 3b, in which the collective motion of two skyrmion clusters can be found.

Title: Supplementary Movie 7:
Description: The movie contains the raw images shown in Supplementary Fig. S8, in which the nonzero skyrmion Hall effect is clearly observed in the low current regime.

Title: Supplementary Movie 8:
Description: The movie contains the raw images shown in Fig. 4, in which the combination of creation, motion and annihilation using current pulses are demonstrated
