# Supplementary material for: Electrical manipulation of skyrmions in a chiral magnet
Source: Nat Commun. 2022 Mar 24;13:1593. doi: 10.1038/s41467-022-29217-4 (PMC8948302; doi:10.1038/s41467-022-29217-4)
Supplement: Supplementary file 2 — Supplementary Information [file 41467_2022_29217_MOESM2_ESM.pdf]

Supplementary information

Electrical manipulation of skyrmions in a chiral magnet

Weiwei Wang[1,2], Dongsheng Song[1,*], Wensen Wei[2], Pengfei Nan[1], Shilei Zhang[3], Binghui Ge[1], Mingliang Tian[2,4,5], Jiadong Zang[6,7,8,*], and Haifeng Du[1,2,4,*]

[1]Institutes of Physical Science and Information Technology, Anhui University, Hefei 230601, China

[2]Anhui Province Key Laboratory of Condensed Matter Physics at Extreme Conditions, High Magnetic Field Laboratory, HFIPS, Anhui, Chinese Academy of Sciences, Hefei, 230031, China

[3]School of Physical Science and Technology, ShanghaiTech University, Shanghai 201210, China

[4]Science Island Branch of Graduate School, University of Science and Technology of China, Hefei, Anhui 230026, China

[5]School of Physics and Materials Science, Anhui University, Hefei, 230601, China

[6]Department of Physics and Astronomy, University of New Hampshire, Durham, New Hampshire 03824, USA

[7]Materials Science Program, University of New Hampshire, Durham, New Hampshire 03824, USA

[8]Institute for Theoretical Physics, University of Cologne, 50937 Cologne, Germany

*Corresponding author:

dsong@ahu.edu.cn, Jiadong.Zang@unh.edu and duhf@hmfl.ac.cn

This Supplementary Information contains the following sections.

Supplementary Fig. S1 -- Schematic procedure of the $Co_8Zn_{10}Mn_2$ micro-device fabrication for *in-situ* Lorentz TEM experiments using the FIB-SEM dual-beam system.

Supplementary Table S1 -- The basic sample information in present work.

Supplementary Fig. S2 -- Skyrmion creation with a notch.

Supplementary Fig. S3 -- Simulation of the skyrmion creation under a current pulse

Supplementary Fig. S4 -- Skyrmion creation in the thermal-dominated regime.

Supplementary Fig. S5 -- Skyrmion deletion at a large current density.

Supplementary Fig. S6 -- Skyrmions aggregation at the normal boundary.

Supplementary Fig. S7 -- The mistrack of the skyrmion cluster motion as a function of the skyrmion sizes.

Supplementary Fig. S8 -- Two skyrmion clusters move simultaneously.

Supplementary Fig. S9 -- Critical current density as a function of pulse width.

Supplementary Fig. S10 -- Disorder induced enhancement of skyrmion Hall effects.

Supplementary Fig. S11 -- Nonzero skyrmion Hall effect at low current density.

Supplementary Fig. S12 -- Electrical manipulation of a skyrmion cluster.

Supplementary Note I -- The enhancement of the skyrmion Hall angle.

Captions for Movies -- 1 to 8.

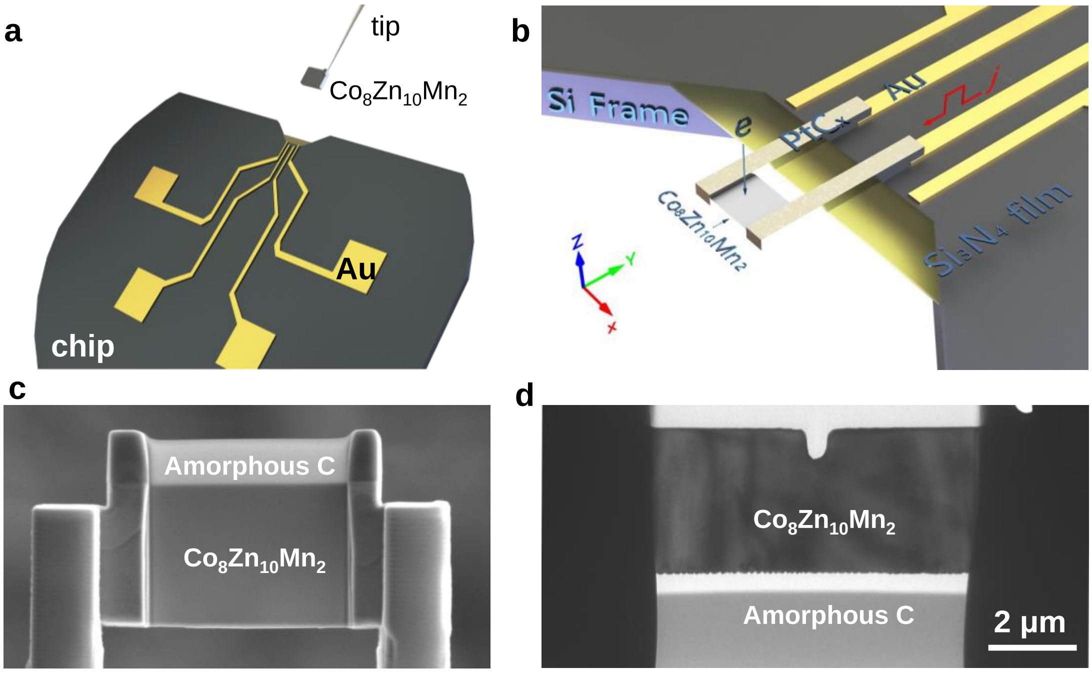

Supplementary Fig. S1 | Schematic procedure of the $Co_8Zn_{10}Mn_2$ micro-device fabrication for *in-situ* Lorentz TEM experiments using the FIB-SEM dual-beam system. a, A thin $Co_8Zn_{10}Mn_2$ lamella with the thickness (*t*) of approximately 1 μm was carved on the surface of bulk $Co_8Zn_{10}Mn_2$ and thereafter transferred to the customized chip with four Au electrodes using the FIB technique. **b**, The lamella was attached to the Au electrodes using ion-beam-induced deposited Pt and then thinned to the desired geometries (t = 140~150 nm in present work). **c,** A typical SEM image of the $Co_8Zn_{10}Mn_2$ micro-device for in-situ Lorentz TEM experiments. **d**, Low magnification TEM image of the $Co_8Zn_{10}Mn_2$ micro-device with a notch.

Tab.S1 | The basic sample information in present work.

Sample Number	Dimensions	Data in Figures
Sample 1	$3200 \times 1400 \times 150\ \mathrm{nm}^3$	Fig.1, Fig.2, Fig.5, Fig.S2, Fig.S4, Fig.S5, Fig.S6, Fig.S12
Sample 2	$5000 \times 3000 \times 140\ \mathrm{nm}^3$	Fig.4, Fig.S9, Fig.S11
Sample 3	$5000 \times 3000 \times 140\ \mathrm{nm}^3$	Fig.3, Fig.S7, Fig.S8

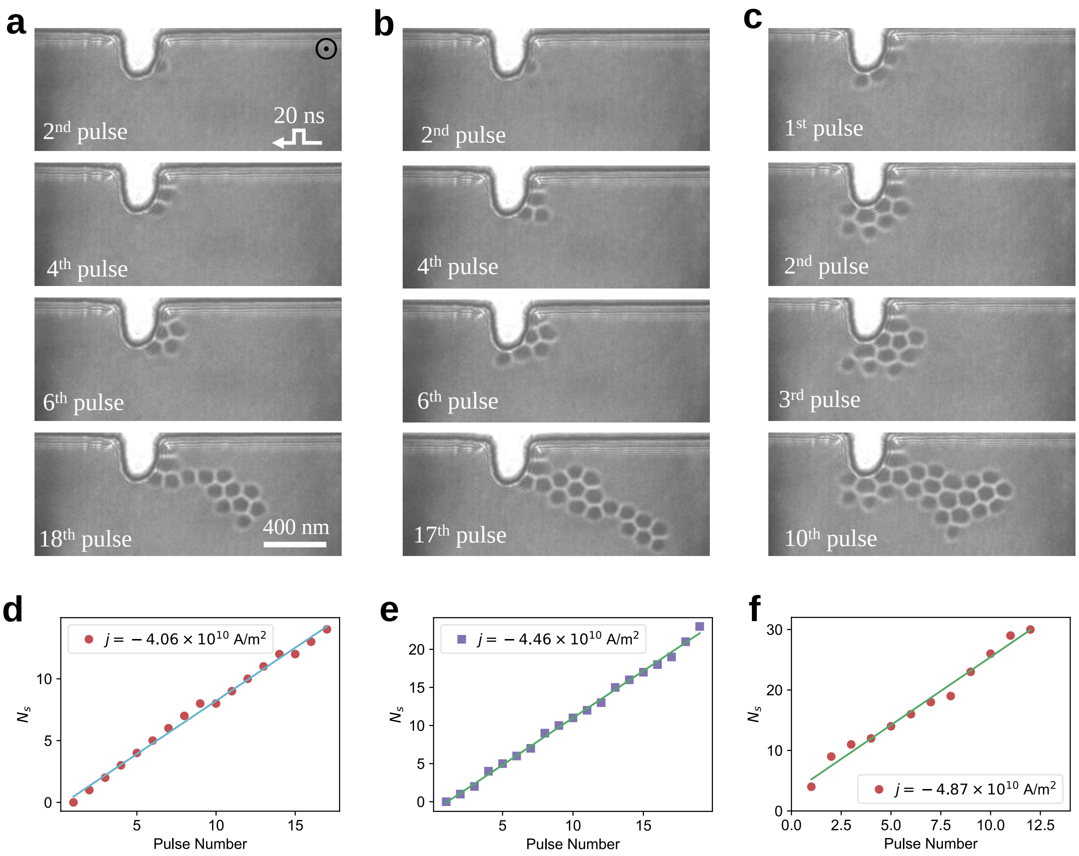

Supplementary Fig. S2 | Skyrmion creation with a notch. a-c, The sequence of Lorentz TEM images of skyrmion creation at $j = -4.06 \times 10^{10}\ \mathrm{A/m^2}$, $j = -4.46 \times 10^{10}\ \mathrm{A/m^2}$ and $j = -4.87 \times 10^{10}\ \mathrm{A/m^2}$, respectively. **d-f**, The number of created skyrmions as a function of applied pulses for these three current densities, as depicted in **a-c**. The linearity between the number of created skyrmion and pulses are observed for various current densities. The scale bar is 400 nm.

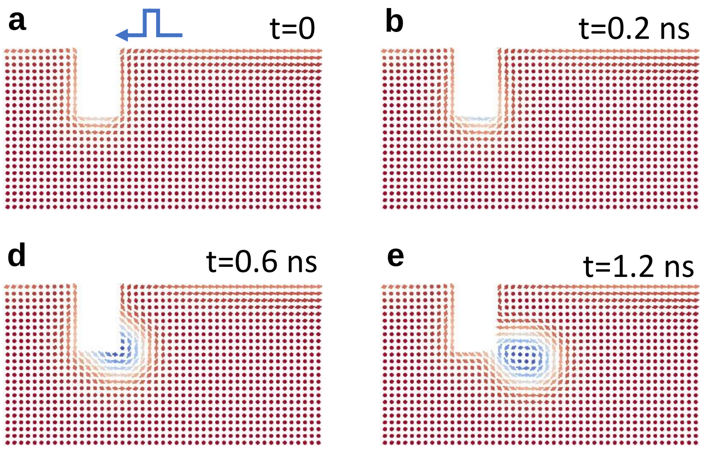

Supplementary Fig. S3 | Simulation of the skyrmion creation under a current pulse. **a-f**, Snapshots of the dynamical magnetization at selected times. A skyrmion with the topological charge of Q = -1 is created on the right side of the notch under a negative j and a positive external field B. In the simulation, u = 50 m/s is used.

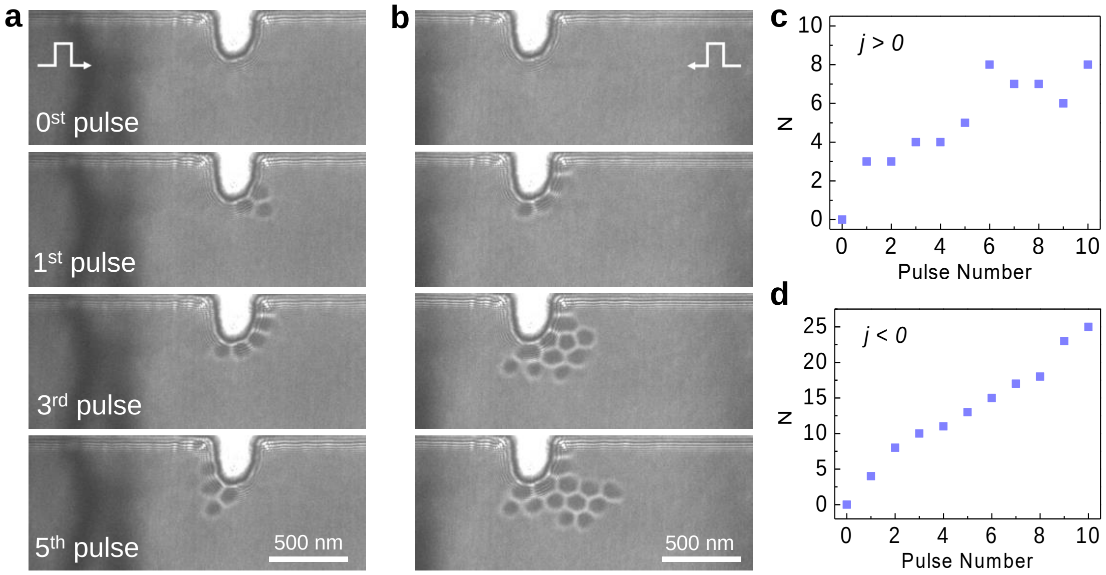

Supplementary Fig. S4 | Skyrmion creation in the thermal-dominated regime. The sequence of Lorentz TEM images of skyrmion creation at $j > 0$ in **a** and $j < 0$ in **b,** respectively**.** The current density is $|j| = 4.87 \times 10^{10}$ A/m^2. Compared with Fig. S2, the positive and negative current pulses both can create magnetic skyrmions, which is the typical feature of the thermal-induced creation of magnetic skyrmions. The number of created skyrmions as a function of the pulse number at $j > 0$ in **c** and $j < 0$ in **d**, respectively. Since the STT effect always exists, the number of created skyrmions at $j > 0$ is much less than that at $j < 0$. The scale bars are 500 nm in **a** and **b**.

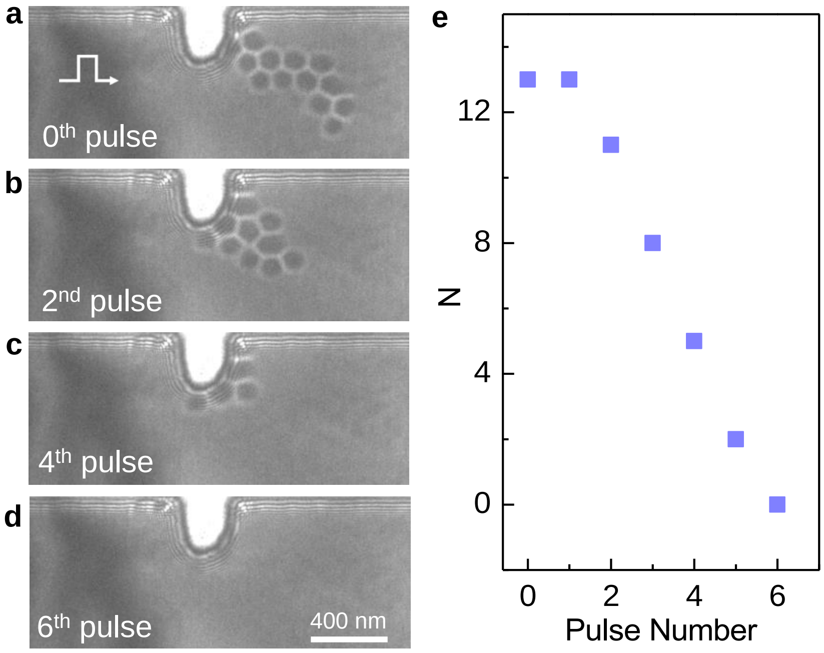

Supplementary Fig. S5 | Skyrmion deletion at a large current density. a-d, The snapshots of skyrmion deletion process by the notch at $j = 4.46 \times 10^{10}\ \mathrm{A/m^2}$ and the pulse width of 20 ns. The external field is B = 70 mT. **a**, Initially, a cluster with 13 skyrmions is located on the right side of the notch. **b**, After applying the first pulse, the skyrmion cluster moved close to the notch with the number unchanged. After that, the number of skyrmions continued to decrease (**c**) and all of the skyrmions are absorbed after the sixth pulse (**d**). **e**, The number of remaining skyrmions as a function of pulse number.

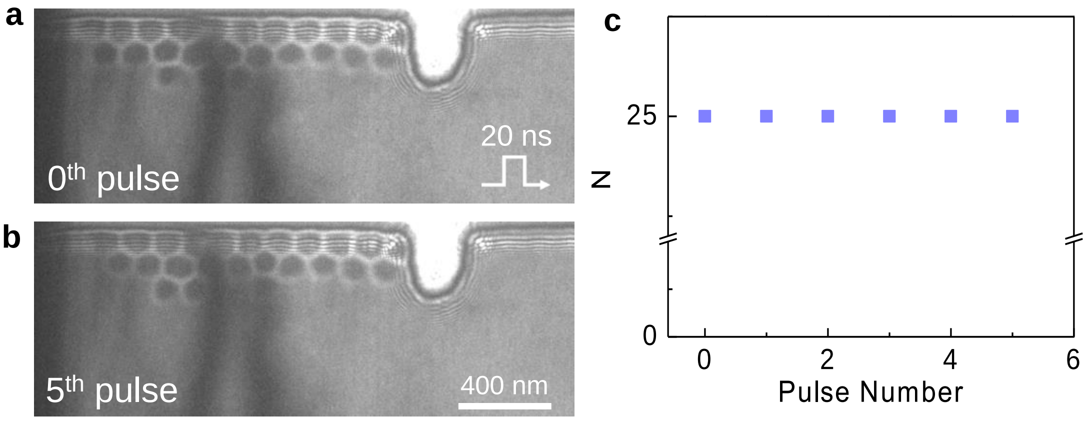

Supplementary Fig. S6 | Skyrmions aggregation at the normal boundary. a, Initially, a skyrmion cluster is aggregated at the normal boundary **b**, After applying five pulses, the shape of the skyrmion cluster changed slightly while its number remains the same, as shown in **c**. The current density is $4.06 \times 10^{10}\ \mathrm{A/m^2}$ and the pulse width is 20 ns, which is the same as that used in Fig. 2 to delete skyrmions. The external magnetic field is $B = 70$ mT. The scale bar is 400 nm.

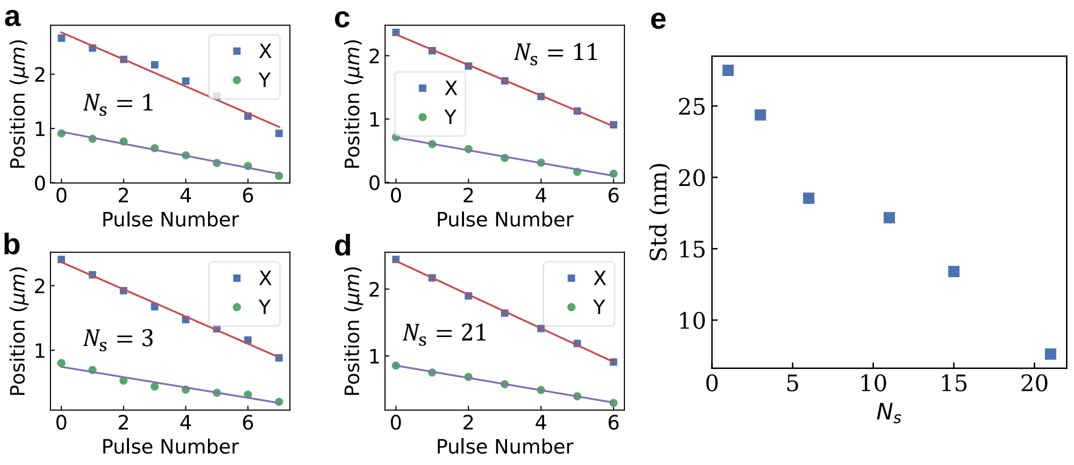

Supplementary Fig. S7 | The mistrack of the skyrmion cluster motion as a function of the skyrmion sizes. a-d, Typical trajectories of skyrmion clusters with different numbers. **e**, The mistrack is defined as the standard deviation between the fitted line and the observed data. The mistrack of the skyrmion motion decreases as the size of cluster increases because both of the thermal fluctuation induced Brownian motion and the pinning related velocity decreases as the skyrmion number increases.

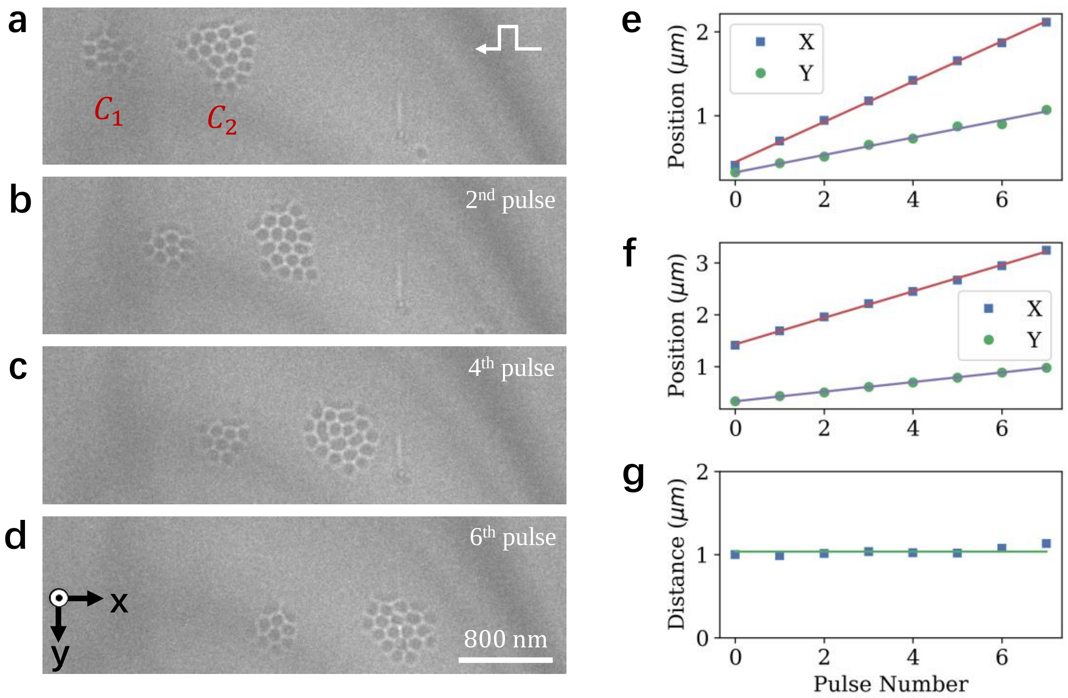

Supplementary Fig. S8 | Two skyrmion clusters move simultaneously. a-d, The snapshots of two skyrmion clusters with $N_s = 11$ and $N_s = 21$ after applying current pulses. The pulse width is 80 ns and the current density is $j = -3.48 \times 10^{10}$ A/m^2. **e-f**, The center (X, Y) of the skyrmion cluster C_1 and C_2 as a function of pulse numbers. **g**, The distance of the two skyrmion clusters as a function of pulse numbers, the distance between two moving skyrmion clusters almost remains a constant. The applied external field is $B = 94$ mT.

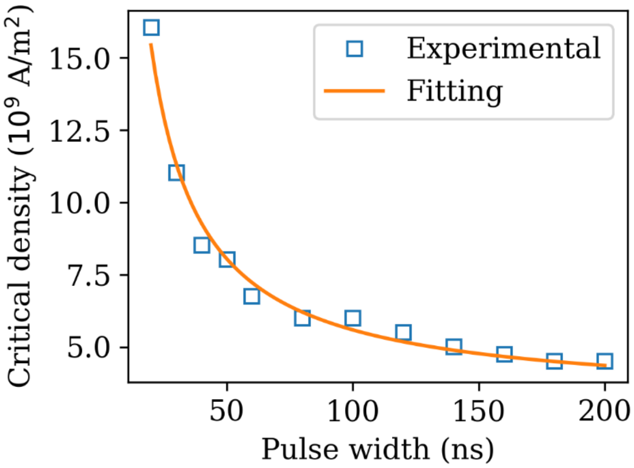

Supplementary Fig. S9 | The critical current density is a function of the pulse width. The critical current density of the skyrmion cluster motion decreases as the pulse width increases.

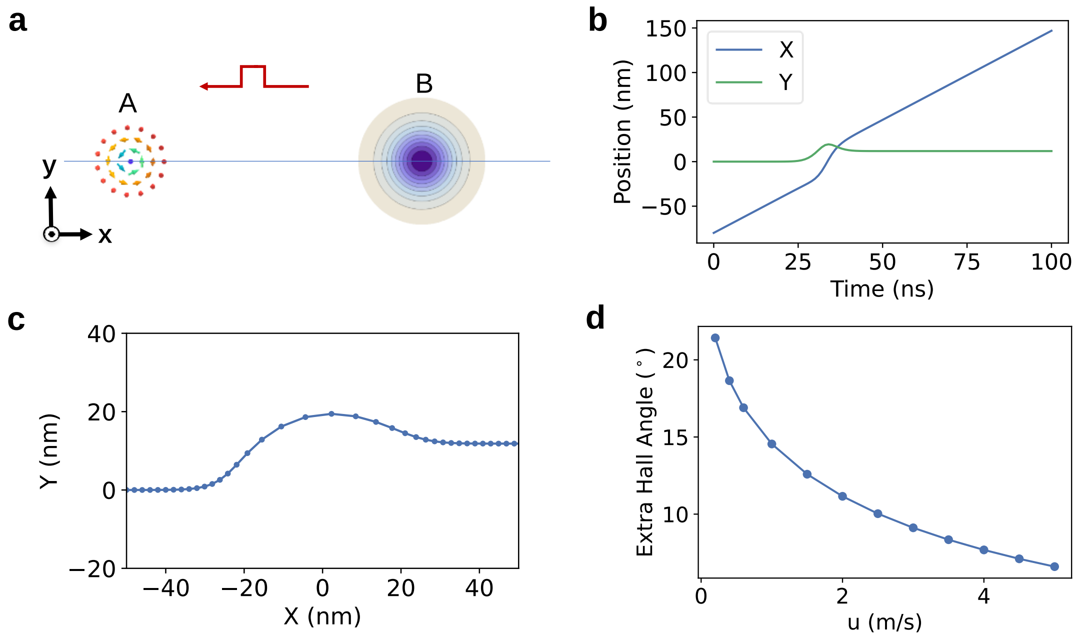

Supplementary Fig. S10 | Disorder induced enhancement of skyrmion Hall effects. a, Illustration of a single skyrmion motion toward a potential arising from local defect. b, The position of the skyrmion as a function of time. The skyrmion position is obtained by numerically solving the Thiele equation [Eq. (S3)] with a Gaussian potential U(x, y) = A $\exp(-r^2/(2\sigma^2))$. The intrinsic hall effect is set to zero, i.e., $\alpha = \beta$, to highlight the influence of the defects on the skyrmion Hall effect. The used parameters are $Q = 1$, $\eta = 1.2$, $\sigma = 10$ nm, $\alpha = 0.1$, $\beta = 0.1$ and $u = 2$ m/s. c, The corresponding trajectory of the skyrmion motion. An extra skyrmion Hall angle is induced by the potential. d, The extra skyrmion Hall angle as a function of u, decreasing with the increase of the current density.

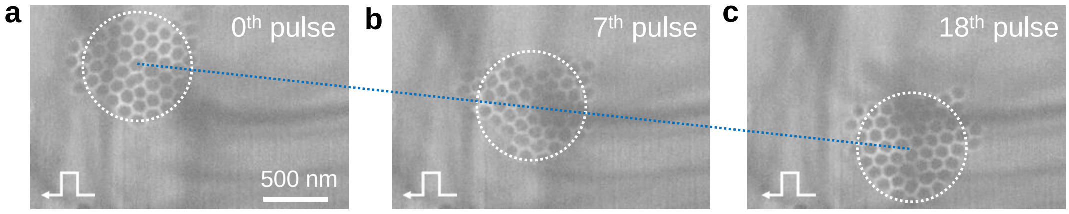

Supplementary Fig. S11 | Nonzero skyrmion Hall effect at low current density. a-c, The snapshots of a skyrmion cluster at $j = -4.5 \times 10^9\ \mathrm{A/m^2}$ and the pulse width of 200 ns. The skyrmion Hall effect is observed even in the low current regime, which is different from the previous studies in Ta/CoFeB/TaO_x heterostructures[1]. The external field is B = 117 mT. The scale bar is 500 nm.

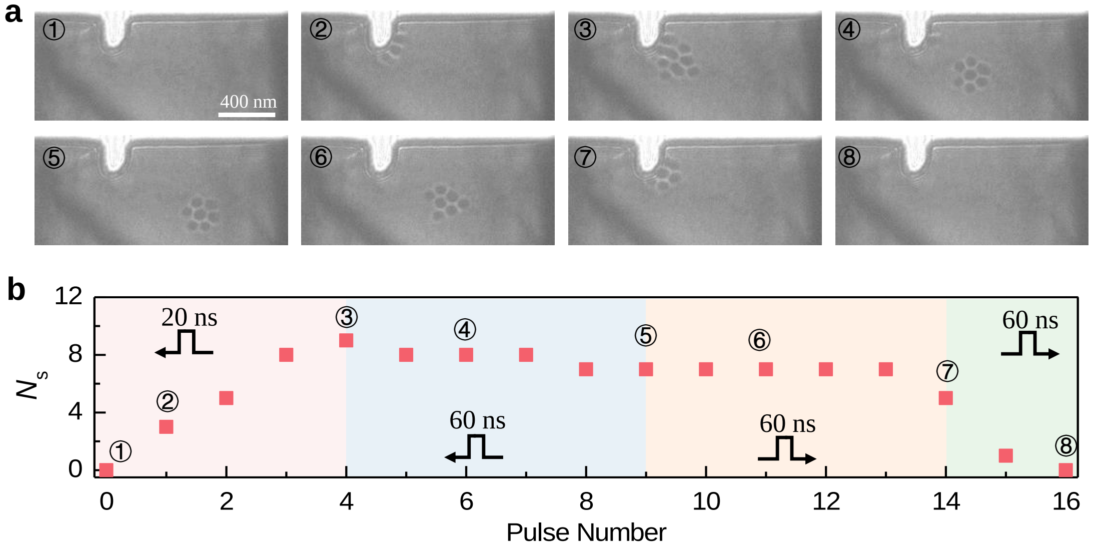

Supplementary Fig. S12 | Electrically manipulation of a skyrmion cluster. a, The snapshots of the skyrmion cluster on different stages. The skyrmion cluster is created in the first stage (①②③) and moves forward in the second stage (③④⑤). The skyrmion then is pushed back on the third stage (⑥) and is finally deleted in the fourth stage (⑦⑧). b, The details of the current pulses and the number of skyrmions as a function of pulse number are plotted. The current densities used on these four stages are $-4.87 \times 10^{10}\ \mathrm{A/m^2}$, $-2.03 \times 10^{10}\ \mathrm{A/m^2}$, $2.03 \times 10^{10}\ \mathrm{A/m^2}$ and $2.03 \times 10^{10}\ \mathrm{A/m^2}$, respectively. The scale bar is 400 nm.

Supplementary Note I – The enhancement of the skyrmion hall angle

The Landau-Lifshitz-Gilbert (LLG) equation including the spin transfer torques (Zhang-Li model) reads[2,3]

$$\frac{\partial \mathbf{m}}{\partial t} = -\gamma \mathbf{m} \times \mathbf{H}_{\text{eff}} + \alpha\, \mathbf{m} \times \frac{\partial \mathbf{m}}{\partial t} + (\mathbf{u} \cdot \boldsymbol{\nabla})\mathbf{m} - \beta[\mathbf{m} \times (\mathbf{u} \cdot \boldsymbol{\nabla})\mathbf{m}] \quad \text{(S1)}$$

where $\mathbf{m}$ is the unit vector of the magnetization, γ is the gyromagnetic ratio, $\mathbf{H}_{\text{eff}}$ is the total effective field, α is the Gilbert damping. The third and fourth terms in the right-hand side of the equation are the spin transfer torques. The parameter u is defined as

$$\mathbf{u} = \frac{gP\mu_{\text{B}}}{2eM_{\text{S}}}\mathbf{j} \quad \text{(S2)}$$

where g is the Landé factor, μ_{B} is the Bohr magneton, e (>0) the electron charge, P the polarization rate of the current, M_{s} is the saturation magnetization and β is the nonadiabatic spin-transfer parameter.

In our experiments, the skyrmion Hall angle is large when the current density is low and it decreases as the current density increases. The enhancement of the skyrmion Hall angle can be attributed to the pinning effects due to disorders or impurities. Starting with the LLG equation including the spin transfer torques (Eq. S1), the Thiele equation can be obtained[4–6]:

$$\mathbf{G} \times (\mathbf{u} + \mathbf{v}) + \mathcal{D}(\beta\mathbf{u} + \alpha\mathbf{v}) = \mathbf{F} \quad \text{(S3)}$$

where $\mathbf{G} = G\mathbf{e}_z$ with $G = 4\pi Q$ describes the Magnus force, $\mathbf{e}_z$ is the unit vector along z axis and Q is the skyrmion number. The second term denotes the dissipative force with $\mathcal{D} = 4\pi\,\eta$ the tensor associated with the shape factor and $\eta_{ij} = (1/4\pi) \int (\partial_i \mathbf{m} \times \partial_j \mathbf{m})\, \mathrm{d}x\, \mathrm{d}y$. For a magnetic skyrmion without distortion $\eta_{ij} = \delta_{ij}\eta$ and typically η is close to unity. The pinning force $\boldsymbol{F}$ originates from the impurity of the material and is given by $F_i = \int \gamma \mathbf{m} \cdot [\partial_i \mathbf{m} \times (\mathbf{m} \times \mathbf{H}_{\text{pin}})]\, \mathrm{d}x\, \mathrm{d}y$. For the current applied in the x-direction, i.e., $\mathbf{u} = (u, 0)$, the solution of the Thiele equation is

$$v_x = -\frac{G^2 + D^2\alpha\beta}{G^2 + \alpha^2 D^2} u + \frac{GF_y + \alpha\, DF_x}{G^2 + \alpha^2 D^2}, \quad \text{(S4)}$$

$$v_y = -\frac{(\alpha - \beta)\, DG}{G^2 + \alpha^2 D^2} u + \frac{\alpha\, DF_y - GF_x}{G^2 + \alpha^2 D^2}. \quad \text{(S5)}$$

Without disorders the solution can be simplified as

$$v_x = -\frac{G^2+D^2\alpha\beta}{G^2+\alpha^2D^2}u, \qquad v_y = -\frac{(\alpha-\beta)\,DG}{G^2+\alpha^2D^2}u \tag{S6}$$

In the limit of $\alpha \ll 1$ and $\beta \ll 1$, the velocities can be simplified further as

$$v_x \approx -b\,j \qquad v_y \approx -\frac{\alpha-\beta}{Q}\eta\,b\,j \tag{S7}$$

where $b = gP\mu_B/2eM_s$. Consequently, the skyrmion Hall angle reads

$$\tan\theta_h = \frac{v_y}{v_x} = \frac{(\alpha-\beta)\,\eta\,Q}{Q^2+\eta^2\alpha\beta} \approx \frac{\eta}{Q}(\alpha-\beta) = \frac{\eta_1}{Q_1}(\alpha-\beta) \tag{S8}$$

where $Q_1 = \pm 1$ and η_1 is the shape factor for a single skyrmion. Note that a single skyrmion and a cluster have the same skyrmion Hall angle, which is because the shape factor η scales linearly with the skyrmion number Q. By ignoring the contribution of the dissipative forces[7], the Eq. (S3) reduces to

$$\mathbf{G} \times (u\,\mathbf{e}_x + \mathbf{v}) = F_v\,\hat{\boldsymbol{v}} + F_\perp \hat{\boldsymbol{v}}_\perp \tag{S9}$$

where the pinning force is split into two components, i.e., the forces parallel to and perpendicular to $\mathbf{v}$. Multiplication $\mathbf{v}$ to Eq. (S9), we obtain

$$\frac{v_y}{v} = \frac{F_v}{4\pi Q}\frac{1}{u} \tag{S10}$$

By assuming that the pinning force only depends on the pinning potential induced by the disorders, the skyrmion Hall angle decreases as the current density increases.

The enhancement of the skyrmion hall angle also can be numerically understood. We consider the situation that a skyrmion moves towards a point-like pinning potential which is axial symmetric, as shown in Fig.S11**a**. The influence of the pinning potential on the skyrmion dynamics are different in two stages. In the first stage ($X < 0$ in Fig.S11**c**) that a skyrmion is approaching to the pinning center, the pinning force biases the skyrmion perpendicularly due to the nonzero Magnus force. In the second stage ($X > 0$ in Fig.S11**c**) that the skyrmion is moving away from the pinning center, the force changes its sign and pulls back the skyrmion. Since the skyrmion is already biased in the first stage, the average force in the second stage is weaker than that in the first stage, leading to a net displacement perpendicular to current direction. This is the physical picture that a pinning potential can enhance the skyrmion Hall angle. At a low current density, a low drift velocity increases the scattering time and thus results in a large skyrmion Hall angle.

Captions for Movies

Supplementary Movie 1: The procedure of the $Co_8Zn_{10}Mn_2$ micro-device fabrication for *in-situ* Lorentz TEM experiments using the FIB-SEM dual-beam system.

Supplementary Movie 2: The movie contains the raw images shown in Fig. 1a, in which the skyrmion creation process after applying the current pulses can be found.

Supplementary Movie 3: The movie contains the raw images shown in Supplementary Fig. S3, in which the skyrmion creation for both negative and positive currents in the thermal-dominated region can be found.

Supplementary Movie 4: The movie contains the raw images shown in Fig. 2a, in which the skyrmion deletion after applying the current pulses can be found.

Supplementary Movie 5: The movie contains the raw images shown in Fig. 3a, in which a single skyrmion with $Q = -1$ is moved using current pulses.

Supplementary Movie 6: The movie contains the raw images shown in Fig. 3b, in which the collective motion of two skyrmion clusters can be found.

Supplementary Movie 7: The movie contains the raw images shown in Supplementary Fig. S8, in which the nonzero skyrmion Hall effect is clearly observed in the low current regime.

Supplementary Movie 8: The movie contains the raw images shown in Fig. 4, in which the combination of creation, motion and annihilation using current pulses are demonstrated.

References

1. Jiang, W. *et al.* Direct observation of the skyrmion Hall effect. *Nat. Phys.* **13**, 162–169 (2017).
2. Zhang, S. & Li, Z. Roles of Nonequilibrium Conduction Electrons on the Magnetization Dynamics of Ferromagnets. *Phys. Rev. Lett.* **93**, 127204 (2004).
3. Thiaville, A., Nakatani, Y., Miltat, J. & Suzuki, Y. Micromagnetic understanding of current-driven domain wall motion in patterned nanowires. *Europhys. Lett. EPL* **69**, 990–996 (2005).
4. Zang, J., Mostovoy, M., Han, J. H. & Nagaosa, N. Dynamics of Skyrmion Crystals in Metallic Thin Films. *Phys. Rev. Lett.* **107**, 136804 (2011).
5. Nagaosa, N. & Tokura, Y. Topological properties and dynamics of magnetic skyrmions. *Nat. Nanotechnol.* **8**, 899–911 (2013).
6. Iwasaki, J., Mochizuki, M. & Nagaosa, N. Universal current-velocity relation of skyrmion motion in chiral magnets. *Nat. Commun.* **4**, 1463 (2013).
7. Everschor, K. *et al.* Rotating skyrmion lattices by spin torques and field or temperature gradients. *Phys. Rev. B* **86**, 054432 (2012).
